# Supplementary material for: Analysis of PPARγ Signaling Activity in Psoriasis
Source: Int J Mol Sci. 2021 Aug 10;22(16):8603. doi: 10.3390/ijms22168603 (PMC8395241; doi:10.3390/ijms22168603)
Supplement: Supplementary file 1 [file ijms-22-08603-s001.zip › Supplemental materials_Analysis of PPARg signaling activity in psoriasis/Pathway models/Models images and html files/Interleukin-17 and interleukin-22 signaling in psoriasis/9327.html]

MAPK8


# Protein MAPK8

|  |  |
| --- | --- |
| URN | urn:agi-llid:5599 |
| Total Entities | 0 |
| Connectivity | 4146 |
| Name | MAPK8 |
| Description | mitogen-activated protein kinase 8 |
| Notes | The protein encoded by this gene is a member of the MAP kinase family. MAP kinases act as an integration point for multiple biochemical signals, and are involved in a wide variety of cellular processes such as proliferation, differentiation, transcription regulation and development. This kinase is activated by various cell stimuli, and targets specific transcription factors, and thus mediates immediate-early gene expression in response to cell stimuli. The activation of this kinase by tumor-necrosis factor alpha (TNF-alpha) is found to be required for TNF-alpha induced apoptosis. This kinase is also involved in UV radiation induced apoptosis, which is thought to be related to cytochrom c-mediated cell death pathway. Studies of the mouse counterpart of this gene suggested that this kinase play a key role in T cell proliferation, apoptosis and differentiation. Several alternatively spliced transcript variants encoding distinct isoforms have been reported. [provided by RefSeq, Apr 2016] |
| Primary Cell Localization | Cytoplasm |
| Class | Protein kinase |

---

|  |  |
| --- | --- |
| Pathway | Proteins Involved in Stem Cell Exhaustion in Aging |
|  | Osteoarthritis |
|  | Skin Aging |
|  | Fatty Acids Block TLR Signaling in Enteroendocrine Cell |
|  | Dopamine Receptors Signaling |
|  | Thrombopoietin Receptors Signaling in Platelet Maturation |
|  | Toll-like Receptors Act through MYD88-TIRAP Signaling |
|  | NOD-like Receptors |
|  | TLR4 Signaling in Leukocytes |
|  | T-Cell Positive Selection and Neglect Induced Death |
|  | T-Cell Central Tolerance |
|  | Scavenger Receptors in Platelet Activation |
|  | Toll-like Receptors in Sterile Inflammation |
|  | MC1R in anti-Inflammatory Signaling |
|  | Basophil Activation |
|  | Eosinophil Survival by Cytokine Signaling |
|  | Mast-Cell Activation via IgE Signaling |
|  | Mast-Cells De Novo Synthesized Mediators via IgE Independent Signaling |
|  | AXL Receptor Inhibits Macrophages and Dendritic Cells Function |
|  | Neutrophil Recruitment and Priming |
|  | Negative Acute Phase Proteins Synthesis |
|  | Positive Acute Phase Proteins Synthesis |
|  | Vascular Endothelial Cell Activation by Cytokines |
|  | Nociception Expression Targets Signaling |
|  | ADRA1A -> IL6 Production |
|  | CNR1/2 -> IL1B/2/4/6/10 Production |
|  | DRD2 Expression Targets |
|  | PTGER2/3 -> Inflammation-Related Expression Targets |
|  | Circadian Clock Genes in Suprachiasmatic Nuclei Neurons |
|  | Apoptosis |
|  | Proteins Involved in Atherosclerosis |
|  | Scavenger Receptor OLR1 in Inflammation Related Endothelial Dysfunction |
|  | Dendritic Cells Function in Atherosclerosis |
|  | Dilated Cardiomyopathy |
|  | Proteins Involved in Dilated Cardiomyopathy |
|  | Proteins Involved in Hypertrophic Cardiomyopathy |
|  | Proteins Involved in Arterial Hypertension |
|  | Myocardial Remodeling |
|  | Myocardial Infarction |
|  | Proteins Involved in Myocardial Ischemia |
|  | Inflammatory Reaction in Acne Vulgaris |
|  | Mast-Cells Activation in Atopic Dermatitis |
|  | Proteins Involved in Melanoma |
|  | IL17 Signaling in Psoriasis |
|  | IL22 Induces Keratinocyte Proliferation in Psoriasis |
|  | Proteins with Altered Expression in Psoriasis |
|  | Dendritic Cells Function in Psoriasis |
|  | beta-Cell Death in Diabetes Mellitus Type 2 |
|  | Proteins Involved in Diabetes Mellitus Type 2 |
|  | Podocyte Dysfunction in Diabetic Nephropathy |
|  | Proteins Involved in Diabetic Nephropathy |
|  | Proteins Involved in Insulin Resistance |
|  | Insulin Resistance in Myocytes Induced by Obesity |
|  | Insulin Resistance in Hepatocytes |
|  | Proteins Involved in non-Alcoholic Fatty Liver Disease |
|  | beta-Cell Destruction through Cytokines in Diabetes Mellitus |
|  | FOXO1 and SREBP-1C Role in beta-Cell Suppression (Rodent Model) |
|  | GNRH Signaling Impairment in Hypogonadotrophic Hypogonadism |
|  | Fatty Acids in Hypertension and Atherosclerosis Development |
|  | Proteins Involved in Obesity |
|  | Macrophage Dysfunction |
|  | OipA Signaling in Helicobacter Pylori Infection |
|  | Dendritic Cell Function in Ulcerative Colitis |
|  | B-Cell Acute Lymphoblastic Leukemia |
|  | B-Cell Chronic Lymphocytic Leukemia |
|  | Chronic Myeloid Leukemia |
|  | Proteins Involved in Chronic Myeloid Leukemia |
|  | Follicular Lymphoma |
|  | Osteoclast Activation in Multiple Myeloma |
|  | Multiple Myeloma |
|  | T-Cell Acute Lymphoblastic Leukemia |
|  | SERPINA1 Associated Liver Damage |
|  | Proteins Involved in Hepatocellular Carcinoma |
|  | Hepatocellular Carcinoma |
|  | Growth Factor Signaling in Hepatocellular Carcinoma |
|  | Pancreatic Ductal Carcinoma |
|  | Growth Factor Signaling in Pancreatic Neoplasms |
|  | Pancreatic Neuroendocrine Tumors |
|  | TGFBR Signaling in Pancreatic Neoplasms |
|  | TSLP Signaling in Bronchial Epithelial Cell in Asthma |
|  | IgE Induces Airway Smooth Muscle Cell Proliferation |
|  | Basophil Activation in Asthma |
|  | Mast-Cell Activation in Asthma |
|  | Mucin Production in Goblet Airway Epithelial Cells |
|  | CFTR Facilitates Oxidative Stress in Airway Epithelium in CF |
|  | Airway Epithelial Cell Dysfunction |
|  | Osteoclast Activation in Gout |
|  | Monocytes Function in Gout |
|  | TNF and IL1B Induce Metalloproteinase Synthesis in Osteoarthritis |
|  | Apoptosis of Chondrocyte |
|  | Dendritic Cell Dysfunction in Psoriatic Arthritis |
|  | Synovial Fibroblast Activation in Psoriatic Arthritis |
|  | Keratinocyte Activation in Psoriatic Arthritis |
|  | Synovial Fibroblast Proliferation in Rheumatoid Arthritis |
|  | Osteoclast Activation in Rheumatoid Arthritis |
|  | Apoptosis Block in Synovial Fibroblast |
|  | TLR2 Induced Synovial Fibroblast Activation in Rheumatoid Arthritis |
|  | Proteins Involved in Rheumatoid Arthritis |
|  | Skin Fibrosis |
|  | CCR5 Signaling in Macrophages |
|  | Apoptosis Block in Infected Cells |
|  | TNF Receptor Associated Periodic Syndrome |
|  | Cryopyrin (NLPR3) Associated Periodic Syndromes |
|  | Majeed Syndrome |
|  | ER Stress in Huntington Disease |
|  | Proteins Involved in Huntington Disease |
|  | IL6 and Insulin Resistance in Muscular Dystrophies |
|  | IL6 Overexpression After Contraction in Skeletal Muscles in Muscular Dystrophies |
|  | Muscular Dystrophies |
|  | Dystrophin Glycoprotein Complex Signaling in Muscular Dystrophies |
|  | Osteoclast Activation in Postmenopause |
|  | Proteins Involved in Alzheimer's Disease |
|  | Glioma Invasion Signaling |
|  | Proteins Involved in Glioma |
|  | Growth Factor Signaling in Neuroblastoma |
|  | Neuroblastoma |
|  | Proteins Involved in Parkinson's Disease |
|  | Proteins Involved in Breast Cancer Related to WNT Signaling Pathway |
|  | Proteins Involved in Breast Cancer Related to ESR1 Signaling Pathway |
|  | WNT Signaling in Breast Cancer |
|  | ESR1/ERBB Positive Luminal Breast Cancer |
|  | ESR1 Signaling in Breast Cancer |
|  | Endometrial Cancer |
|  | Endometrioid Endometrial Cancer |
|  | Clear Cell Endometrial Cancer and Papillary Serous Endometrial Cancer |
|  | Proteins Involved in Endometriosis |
|  | Angiogenesis in Endometriosis |
|  | Proteins Involved in High-Grade Serous Ovarian Carcinoma |
|  | Endometrioid Ovarian Carcinoma |
|  | Proteins Involved in Endometrioid Ovarian Carcinoma |
|  | Clear Cell Ovarian Carcinoma |
|  | Proteins Involved in Mucinous Ovarian Carcinoma |
|  | High-Grade Serous Ovarian Carcinoma |
|  | Low-Grade Serous Ovarian Carcinoma |
|  | Proteins Involved in Low-Grade Serous Ovarian Carcinoma |
|  | Ovarian Cancer |
|  | Proteins Involved in Clear Cell Ovarian Carcinoma |
|  | Mucinous Ovarian Carcinoma |
|  | Subepithelial Fibroblasts in Middle Ear Cholesteatoma |
|  | HPV E5 Regulates EDNRA and EGFR Signaling Pathway in Keratinocytes |
|  | Androgen Receptor/Akt Signaling |
|  | RNase L Signaling in Prostate Cancer |
|  | Proteins Involved in Age-Related Macular Degeneration |
|  | ER Stress in Macular Degeneration |
|  | Steroids Induced Cataract |
|  | MYOC Associated Glaucoma |
|  | Proteins Involved in Noise-Induced Hearing Loss |
|  | Aminoglycosides and Cisplatin Induced Ototoxicity (Mouse Model) |
|  | Proteins Involved in Hearing Loss |
|  | Proteins Involved in Otitis Media |
|  | Cytokines Trigger Otitis Media |
|  | Immune System Role in Otitis Media |
|  | Alveolar Macrophage Negative Regulation Declined in COPD |
|  | Alveolar Macrophages Dysfunction in COPD |
|  | Proteins Involved in Chronic Obstructive Pulmonary Disease |
|  | T-Cell Cytotoxic Activation |
|  | Mucin Hyperproduction in Goblet and Mucous Cells |
|  | WNT Signaling in Cystic Kidney Disease |
|  | Polycystins Mutation Effects in Cystic Kidney Disease |
|  | Proteins Involved in Polycystic Kidney Disease |
|  | Proteins Involved in Glomerulonephritis |
|  | Podocyte Dysfunction in Glomerulonephritis |
|  | Apoptosis of Renal Tubule Epithelial Cell in Pyelonephritis |
|  | ANGPT2 -> AP-1/CREBBP/MYC Expression Targets |
|  | AREG -> AP-1 Expression Targets |
|  | Dopamine/Gi Expression Targets |
|  | BTC -> AP-1/ATF/CREB Expression Targets |
|  | Frizzled Receptors -> ARRB1/ARRB2 non-Canonical Signaling |
|  | Complement Component GPCRs Signaling |
|  | EGF -> AP-1/ATF Expression Targets |
|  | EGF -> CREB/CREBBP/ELK/SRF/MYC Expression Targets |
|  | EGF -> TP53 Expression Targets |
|  | EGFR/ERBB2 -> TP53 Signaling |
|  | EGFR -> AP-1/ATF2 Signaling |
|  | EGFR -> AP-1/CREB/ELK/SRF/MYC Signaling |
|  | EREG -> AP-1/ATF Expression Targets |
|  | Erythropoietin -> AP-1/MYC/CREB Expression Targets |
|  | F2 -> AP-1/CREB/ELK/SRF/SP1 Expression Targets |
|  | PLG -> AP-1/CREB/ELK/SRF/SP1 Expression Targets |
|  | FGF9 -> AP-1/CREB/MYC Expression Targets |
|  | FGF10 -> AP-1/CREB/CREBBP/MYC Expression Targets |
|  | FGF1 -> AP-1/CREB/ELK/SRF/MYC Expression Targets |
|  | FGF8 -> AP-1/CREB/MYC Expression Targets |
|  | FGF18 -> AP-1/CREB Expression Targets |
|  | FGF4 -> AP-1/MYC Expression Targets |
|  | FGF7 -> AP-1/CREB/CREBBP/MYC Expression Targets |
|  | FGF2 -> AP-1/CREB/CREBBP/ELK/SRF/MYC Expression Targets |
|  | FGFR -> AP-1/CREB/CREBBP/ELK/SRF/MYC Signaling |
|  | FrizzledR -> JUN/PAX2 Signaling |
|  | HBEGF -> AP-1/ATF Expression Targets |
|  | HBEGF -> TP53 Expression Targets |
|  | HBEGF -> CREB/MYC Expression Targets |
|  | HGF -> AP-1/CREB/ELK/SRF/MYC Expression Targets |
|  | HGFR -> AP-1/CREB/MYC Signaling |
|  | RAS/RAF/MAPK Signaling Activation in Cancer |
|  | RAS/RAF/MAPK Signaling Activation by Blocking of Tumor Suppressors |
|  | Apoptosis Block by Death Receptor Signaling in Cancer |
|  | Apoptosis Evasion in Cancer: Overview |
|  | Apoptosis Block by BCL2 Family Proteins in Cancer |
|  | VEGFA Dependent Angiogenesis in Cancer |
|  | Inhibition of THBS1 Activates Angiogenesis in Cancer |
|  | VEGF Independent Angiogenesis in Cancer |
|  | Hyaluronic Acid, CD44 and HMMR in Cancer Cell Invasion and Survival |
|  | FibronectinR -> AP-1/ELK/SRF/SREBF Signaling |
|  | CTGF -> AP-1/CREB/MYC Expression Targets |
|  | PAF/Gq -> AP-1/ATF1/CREB/ERK/SRF Expression Targets |
|  | Dronabinol/Anandamide Expression Targets |
|  | S1P Expression Targets |
|  | GPCRs Family -> Expression Targets in Lymphoid System and Blood |
|  | NRG1 -> CREB/CREBBP/ELK/SRF/MYC Expression Targets |
|  | NRG1 -> TP53 Expression Target |
|  | NRG1 -> AP-1/ATF Expression Targets |
|  | NGF -> SMAD3/NF-kB Expression Targets |
|  | NGF -> AP-1/TP53/MYC Expression Targets |
|  | BDNF Expression Targets |
|  | NTF3 Expression Targets |
|  | NTF4 Expression Targets |
|  | NOTCH -> TCF3 Signaling |
|  | NOTCH Expression Targets |
|  | PDGFB -> AP-1/CREB/MYC Expression Target |
|  | PDGFD -> AP-1 Expression Targets |
|  | PDGF -> AP-1/CREB/CREBBP/MYC Expression Targets |
|  | PDGFR -> AP-1/MYC Signaling |
|  | AVP/Gs -> CREB/ELK/SRF/AP-1/EGR Expression Targets |
|  | GNRH1 Expression Targets |
|  | GNRH2 Expression Targets |
|  | EDN1 Expression Targets |
|  | EDN3 Expression Targets |
|  | AVP/Gq -> CREB/ELK/SRF/AP-1/EGR Expression Targets |
|  | AngiopoietinR -> AP-1 Signaling |
|  | GDNF -> HSF1 Signaling |
|  | EphrinB -> MAPK/JUN/FOS Signaling |
|  | NTRK -> AP-1/CREB/ELK/SRF/MYC/SMAD3/TP53 Signaling |
|  | GAS6 -> AP-1/CREB Expression Targets |
|  | FIGF -> AP-1 Expression Target |
|  | KITLG -> AP-1/CREB/CREBBP/MYC Expression Targets |
|  | GDNF -> HSF1 Expression Targets |
|  | CSF1 -> AP-1/CREB/CREBBP/MYC Expression Targets |
|  | PGF -> AP-1/CREB/CREBBP/MYC Expression Targets |
|  | FLT3LG -> AP-1/CREB/CREBBP Expression Targets |
|  | TGFBR -> AP-1 Signaling |
|  | TNF-alpha/TNFRSF1A Signaling |
|  | JNK/MAPK Signaling |
|  | AXL Receptor Tyrosine Kinase Signaling |
|  | VEGF Signaling |
|  | EGFR Signaling |
|  | WNT Planar Cell Polarity (PCP) non-Canonical Signaling |
|  | HRAS Signaling |
|  | TGFA -> TP53 Expression Targets |
|  | TGFA/AP-1/ATF Expression Targets |
|  | TGFA -> CREB/CREBBP/ELK-SRF/MYC Expression Targets |
|  | TGFB3-TGFBR2 Expression Targets |
|  | TGFB2-TGFBR2 Expression Targets |
|  | TGFB1-TGFBR2 Expression Targets |
|  | FASLG Expression Targets |
|  | TNFSF10 Expression Targets |
|  | LTA Expression Targets |
|  | TNFSF14 Expression Targets |
|  | TNF -> AP-1 Expression Targets |
|  | CD72 -> AP-1 Expression Targets |
|  | CD40LG -> NF-kB/ELK/SRF -> CREB/NFATC Expression Targets |
|  | TNFSF13 Expression Targets |
|  | TNFSF13B Expression Targets |
|  | TNF -> ELK-SRF Expression Targets |
|  | TNF -> TP53/ATF Expression Targets |
|  | EDA Expression Targets |
|  | EctodysplasinR -> AP-1 Signaling |
|  | TNFR -> CREB/ELK-SRF Signaling |
|  | TNFR -> AP-1/ATF/TP53 Signaling |
|  | NGFR -> AP-1/CEBPB/CREB/ELK/SRF/TP53 Signaling |
|  | TNFRSF6 -> DDIT3 Signaling |
|  | TNFRSF6 -> HSF1 Signaling |
|  | TNFRSF1A -> AP-1/ATF/TP53 Signaling |
|  | TNFRSF1A -> CREB/ELK-SRF Signaling |
|  | Thrombopoietin -> SPI1 Expression Targets |
|  | Thrombopoietin -> AP-1/CREB/CREBBP/MYC Expression Targets |
|  | TLR4 -> AP-1 Expression Targets |
|  | TLR5 Expression Targets |
|  | TLR7 Expression Targets |
|  | TLR4 -> AP-1/EGR1/HIF1A Expression Targets |
|  | IL1B -> PGE2 Expression Targets |
|  | TLR1 -> 2/6 Expression Targets |
|  | TLR9 Expression Targets |
|  | TLR -> AP-1 Signaling |
|  | ROS in Angiotensin Mediated Cardiovascular Remodeling and Hypertrophy |
|  | VEGFA -> AP-1/CREBBP/MYC Expression Targets |
|  | VEGFR -> AP-1/CREB/MYC Signaling |
|  | WNT1 Expression Targets |
|  | WNT3A Expression Targets |
|  | WNT4 Expression Targets |
|  | WNT5A Expression Targets |
|  | WNT7A Expression Targets |
|  | 1\_Inflammation in keratinocytes in the pathology of acne |
|  | 2\_Acute phase initiation in atopic dermatitis |
|  | 2\_Interleukin-17 and interleukin-22 signaling in psoriasis |
|  | 1\_TNF and IL1B provoke ECM degradation in osteoarthritis |
|  | 1\_Osteoclast-mediated bone resorption in postmenopausal women |
|  | 1\_Formation of cysts in polycystic kidney disease |
|  | 2\_Podocyte dysfunction in glomerulonephritis |
|  | 1\_Disruption of luteinizing hormone and follicle-stimulating hormone secretion in PCOS |
|  | 2\_MYOC-associated glaucoma |
|  | 3\_Endoplasmic reticulum stress in age-related macular degeneration |
|  | 2\_Pathogens stimulate mucins expression in the middle ear |
|  | 2\_Cigarette smoke and inflammation cause alveolar epithelial cell death in COPD |
|  | 3\_Role of alveolar macrophages in pulmonary tissue destruction in COPD |
|  | 2\_Intestine epithelial cells drive immunological responses in ulcerative colitis |
|  | 2\_Lipotoxicity in NAFLD |
|  | 6\_p12I Viral protein promotes T-cell activation in HTLV-1 infection |
|  | 4\_2\_Insulin resistance: inflammation-related insulin resistance |
|  | 4\_3\_Insulin resistance: FFA-related insulin resistance |
|  | 2\_SERPINA1-associated liver damage |
|  | 2\_Neuron and Schwann cell death |
|  | HRAS->RAF->MAPK1/3 |
|  | mapk1 protein modificators |
|  | Interleukin-17 and interleukin-22 signaling in psoriasis |

---

|  |  |
| --- | --- |
| Group | Genes with Mutations Associated with Ageing (non-Mammalian Animal Model) |

---

|  |  |
| --- | --- |
| Source | GO component |
|  | ResNet |
|  | curated |

---

|  |  |
| --- | --- |
| MedScan ID | 5599 |

---

|  |  |
| --- | --- |
| LocusLink ID | 5599 |
|  | 26419 |
|  | 116554 |
|  | 71973 |
|  | 539941 |
|  | 19098 |
|  | 105560 |
|  | 445436 |
|  | 326419 |
|  | 65236 |
|  | 50656 |
|  | 102478 |

---

|  |  |
| --- | --- |
| Alias | OTTMUSP00000019791 |
|  | c-Jun N-terminal kinase 1 |
|  | SAPK1/JNK |
|  | p46JNK1 |
|  | OTTHUMP00000019558 |
|  | p54 gamma |
|  | OTTHUMP00000019556 |
|  | MAP kinase 8 |
|  | stress-activated protein kinase JNK I |
|  | mitogen-activated protein kinase 8 isoform JNK1 beta2 |
|  | Jun NH2-terminal kinase I |
|  | AI849689 |
|  | protein kinase JNK1 |
|  | protein kinase JNK I |
|  | stress-activated protein kinase JNK1 |
|  | JNK1A2 |
|  | stress-activated protein kinase 1 |
|  | JNK21B1/2 |
|  | OTTHUMP00000019552 |
|  | JNK1 beta protein kinase |
|  | mitogen activated protein kinase 8 |
|  | SAPK I |
|  | SAPK1 |
|  | JNK I |
|  | Jun NH2-terminal kinase 1 |
|  | JNK1 |
|  | stress-activated protein kinase 1c |
|  | PRKM8 |
|  | OTTMUSP00000019794 |
|  | JNK1 alpha2 protein kinase |
|  | mitogen-activated protein kinase 8 isoform JNK1 alpha1 |
|  | cJun N-terminal kinase 1 |
|  | c-Jun NH2-kinase 1 |
|  | c-Jun NH2-terminal kinase 1 |
|  | PIR:A53063 |
|  | OTTMUSP00000019790 |
|  | mitogen activated protein kinase VIII |
|  | SAPK gamma |
|  | OTTMUSP00000019793 |
|  | OTTHUMP00000019555 |
|  | JNK1 beta1 protein kinase |
|  | c-Jun NH2-terminal kinase I |
|  | protein kinase mitogen-activated 8 |
|  | MAPK8 |
|  | JNK-46 |
|  | protein kinase mitogen-activated VIII |
|  | SAPK1/c-Jun N-terminal kinase |
|  | JNK1 alpha protein kinase |
|  | SAPK1c |
|  | stress activated protein kinase 1 |
|  | c-Jun N-terminal kinase I |
|  | JNK |
|  | JUN N-terminal kinase |
|  | mitogen-activated protein kinase 8 |
|  | MAPK 8 |
|  | c-jun NH2-terminal kinase |
|  | Jun NH2-terminal kinase |
|  | c-Jun N-terminal kinase |
|  | c-Jun NH2-kinase |
|  | cJun N-terminal kinase |
|  | Stress-activated protein kinase JNK1 |
|  | Stress-activated protein kinase JNK I |
|  | RBPMS2 |
|  | JNK1alpha2 |
|  | expressed sequence AI849689 |
|  | RBPMS2s |
|  | JNK1-alpha-1 |
|  | RNA-binding protein with multiple splicing II |
|  | similar to RIKEN cDNA 2400008B06 |
|  | expressed sequence ai316523 |
|  | Pristane induced arthritis QTL 1 |
|  | Pristane induced arthritis QTL I |
|  | mitogen-activated protein kinase 8 transcript variant II |
|  | Pia I |
|  | mitogen-activated protein kinase 8 transcript variant 2 |
|  | Pia1 |
|  | RNA-binding protein with multiple splicing 2 |
|  | scaffold |
|  | chromosome |
|  | zgc:112379 |

---

|  |  |
| --- | --- |
| EC Number | 2.7.11.24 |
|  | 2.7.1.- |
|  | 2.7.11.1 |
|  | 2.7.1.37 |

---

|  |  |
| --- | --- |
| GO ID | 0005524 |
|  | 0004705 |
|  | 0004707 |
|  | 0019899 |
|  | 0042826 |
|  | 0035033 |
|  | 0016301 |
|  | 0004674 |
|  | 0038095 |
|  | 0007254 |
|  | 0007258 |
|  | 0034198 |
|  | 0071276 |
|  | 0071345 |
|  | 0071222 |
|  | 0071260 |
|  | 0071310 |
|  | 0034614 |
|  | 0035556 |
|  | 0043066 |
|  | 0032091 |
|  | 0048666 |
|  | 0018105 |
|  | 0018107 |
|  | 0043065 |
|  | 0031281 |
|  | 0090045 |
|  | 0010628 |
|  | 1900740 |
|  | 0051247 |
|  | 0006468 |
|  | 1902595 |
|  | 0051090 |
|  | 0042752 |
|  | 0010468 |
|  | 0031063 |
|  | 0016241 |
|  | 0032880 |
|  | 0009411 |
|  | 0009612 |
|  | 0048511 |
|  | 0051403 |
|  | 0030424 |
|  | 0097441 |
|  | 0005737 |
|  | 0005829 |
|  | 0005739 |
|  | 0043005 |
|  | 0005654 |
|  | 0005634 |
|  | 0019894 |
|  | 0000166 |
|  | 0004672 |
|  | 0004712 |
|  | 0016740 |
|  | 0070301 |
|  | 0071732 |
|  | 0048813 |
|  | 0048263 |
|  | 0006954 |
|  | 0001764 |
|  | 0031175 |
|  | 0097150 |
|  | 0001503 |
|  | 0016310 |
|  | 0045740 |
|  | 2001235 |
|  | 0010666 |
|  | 0030335 |
|  | 2000017 |
|  | 0034352 |
|  | 0031116 |
|  | 0002052 |
|  | 0043525 |
|  | 2001224 |
|  | 0071803 |
|  | 0031398 |
|  | 1901485 |
|  | 0097300 |
|  | 0061833 |
|  | 0046605 |
|  | 0045664 |
|  | 0006355 |
|  | 0046686 |
|  | 0009408 |
|  | 0042542 |
|  | 0006970 |
|  | 0007165 |
|  | 0097050 |
|  | 0032839 |
|  | 0043204 |
|  | 0002102 |
|  | 0031982 |
|  | 0000165 |
|  | 1904646 |
|  | 0071347 |
|  | 0034349 |
|  | 0048545 |
|  | 0006950 |
|  | 0071216 |
|  | 0005622 |
|  | 0002755 |
|  | 0002756 |
|  | 0035666 |
|  | 0006915 |
|  | 0097190 |
|  | 0045087 |
|  | 0097193 |
|  | 0048011 |
|  | 0034166 |
|  | 0034134 |
|  | 0034138 |
|  | 0034142 |
|  | 0034146 |
|  | 0034162 |
|  | 0038123 |
|  | 0038124 |
|  | 0002224 |
|  | 0016772 |
|  | 0008063 |
|  | 0008633 |
|  | 0034130 |
|  | 0023014 |
|  | 0033267 |
|  | 0008624 |
|  | 0008629 |
|  | 0031558 |
|  | 0008152 |
|  | 0005515 |
|  | 0006928 |
|  | 0046933 |
|  | 0004682 |
|  | 0050775 |
|  | 0016469 |
|  | 0004713 |
|  | 0046961 |
|  | 0004691 |
|  | 0015986 |
|  | 0003723 |
|  | 0003676 |

---

|  |  |
| --- | --- |
| KEGG ID | hsa:5599 |
|  | mmu:26419 |
|  | rno:116554 |

---

|  |  |
| --- | --- |
| MedLine Reference | 16491099 |
|  | 16938889 |
|  | 12869527 |
|  | 12213570 |
|  | 11927625 |
|  | 16412424 |
|  | 12135322 |
|  | 16458303 |
|  | 15642137 |
|  | 17008315 |
|  | 11927626 |
|  | 12189144 |
|  | 12651633 |
|  | 7839144 |
|  | 15326485 |
|  | 12122119 |
|  | 15527495 |
|  | 12413764 |
|  | 12971962 |
|  | 9343414 |
|  | 17526490 |
|  | 17365404 |
|  | 12534344 |
|  | 12447443 |
|  | 16571730 |
|  | 15375216 |
|  | 12466851 |
|  | 12189133 |
|  | 12130649 |
|  | 16889766 |
|  | 14967141 |
|  | 14769808 |
|  | 15238629 |
|  | 14766760 |
|  | 15767678 |
|  | 11217851 |
|  | 10781376 |
|  | 8632909 |
|  | 16818881 |
|  | 14551213 |
|  | 16328781 |
|  | 16237070 |
|  | 17300814 |
|  | 10773432 |
|  | 15528206 |
|  | 12015964 |
|  | 15572687 |
|  | 12478662 |
|  | 9516415 |
|  | 16973441 |
|  | 12514174 |
|  | 15657352 |
|  | 11562351 |
|  | 11042694 |
|  | 16579967 |
|  | 17202416 |
|  | 14704277 |
|  | 14733915 |
|  | 14983993 |
|  | 17534150 |
|  | 15041276 |
|  | 9872331 |
|  | 15314024 |
|  | 17519787 |
|  | 14576193 |
|  | 12954641 |
|  | 12445686 |
|  | 11448159 |
|  | 15123655 |
|  | 16321971 |
|  | 17276034 |
|  | 11884395 |
|  | 15836771 |
|  | 16440308 |
|  | 12376563 |
|  | 15696159 |
|  | 15302844 |
|  | 12629045 |
|  | 16949031 |
|  | 15013949 |
|  | 12242293 |
|  | 12296995 |
|  | 16260609 |
|  | 15309413 |
|  | 16543147 |
|  | 15761494 |
|  | 11931768 |
|  | 16081685 |
|  | 12943527 |
|  | 16465391 |
|  | 12244175 |
|  | 12917434 |
|  | 12842852 |
|  | 15665513 |
|  | 10797012 |
|  | 12842862 |
|  | 11700324 |
|  | 12393629 |
|  | 16804062 |
|  | 17298982 |
|  | 16176806 |
|  | 12492400 |
|  | 15755722 |
|  | 16038800 |
|  | 16157589 |
|  | 14512437 |
|  | 12883833 |
|  | 10523642 |
|  | 11307167 |
|  | 16466687 |
|  | 11042159 |
|  | 16092944 |
|  | 16815888 |
|  | 11812769 |
|  | 14765123 |
|  | 16336220 |
|  | 17317777 |
|  | 16607699 |
|  | 17054907 |
|  | 15545623 |
|  | 14985505 |
|  | 1748630 |
|  | 16260419 |
|  | 11323415 |
|  | 11489891 |
|  | 15334056 |
|  | 11466342 |
|  | 11062067 |
|  | 16446354 |
|  | 14707549 |
|  | 11606564 |
|  | 9621077 |
|  | 17333127 |
|  | 16282329 |
|  | 12859962 |
|  | 15358865 |
|  | 7642542 |
|  | 11279197 |
|  | 12130508 |
|  | 10597297 |
|  | 14532003 |
|  | 12503078 |
|  | 9207092 |
|  | 12847227 |
|  | 14499647 |
|  | 8177321 |
|  | 12668503 |
|  | 9030579 |
|  | 15769735 |
|  | 16514068 |
|  | 15731347 |
|  | 9096336 |
|  | 14563325 |
|  | 17015619 |
|  | 16807293 |
|  | 12161171 |
|  | 15474087 |
|  | 11788583 |
|  | 16645635 |
|  | 10498280 |
|  | 12397601 |
|  | 16311603 |
|  | 17608927 |
|  | 8889548 |
|  | 16105650 |
|  | 12661038 |
|  | 11749722 |
|  | 12354774 |
|  | 16085647 |
|  | 17325051 |
|  | 10913135 |
|  | 10471813 |
|  | 16814421 |
|  | 11260070 |
|  | 16687575 |
|  | 10995439 |
|  | 15772134 |
|  | 15528994 |
|  | 10772775 |
|  | 16507367 |
|  | 12700162 |
|  | 16099428 |
|  | 17061159 |
|  | 9129988 |
|  | 17108173 |
|  | 14557276 |
|  | 16794185 |
|  | 16971530 |
|  | 9732264 |
|  | 12039864 |
|  | 14500675 |
|  | 16491125 |
|  | 10912793 |
|  | 12763138 |
|  | 12058026 |
|  | 12853963 |
|  | 15483136 |
|  | 15466414 |
|  | 12058028 |
|  | 12161751 |
|  | 11283254 |
|  | 10551811 |
|  | 16483929 |
|  | 11157052 |
|  | 11278789 |
|  | 12079429 |
|  | 16381010 |
|  | 15673436 |
|  | 16973625 |
|  | 17410600 |
|  | 15316358 |
|  | 15778365 |
|  | 17079291 |
|  | 12592382 |
|  | 14699155 |
|  | 16469705 |
|  | 17229766 |
|  | 16314504 |
|  | 12351658 |
|  | 16802349 |
|  | 15757900 |
|  | 12095152 |
|  | 17041759 |
|  | 16927023 |
|  | 17068110 |
|  | 14633134 |
|  | 16774848 |
|  | 9393873 |
|  | 12091388 |
|  | 12477932 |
|  | 15655348 |
|  | 10349636 |
|  | 14688370 |
|  | 15516492 |
|  | 15797868 |
|  | 15820682 |
|  | 15632024 |
|  | 17555943 |
|  | 17283057 |
|  | 9195981 |
|  | 14712227 |
|  | 14578290 |
|  | 16243842 |
|  | 15254243 |
|  | 12376107 |
|  | 11350938 |
|  | 11278799 |
|  | 10559486 |
|  | 10747973 |
|  | 15837794 |
|  | 11432831 |
|  | 12970181 |
|  | 17015265 |
|  | 10629060 |
|  | 15260977 |
|  | 17003331 |
|  | 15272135 |
|  | 15637062 |
|  | 17496921 |
|  | 8654373 |
|  | 12171600 |
|  | 12591950 |
|  | 14637155 |
|  | 16687404 |
|  | 17284179 |
|  | 8621542 |
|  | 16824735 |
|  | 15981086 |
|  | 11238452 |
|  | 8917518 |
|  | 11406125 |
|  | 11076861 |
|  | 11602244 |
|  | 17303384 |
|  | 16153436 |
|  | 17337450 |
|  | 12140754 |
|  | 15711095 |
|  | 15890690 |
|  | 10230788 |
|  | 10391943 |
|  | 17481915 |
|  | 12351702 |
|  | 12665585 |
|  | 14504284 |
|  | 15689551 |
|  | 17045576 |
|  | 9788880 |
|  | 12933347 |
|  | 17074809 |
|  | 17079781 |
|  | 16895791 |
|  | 17192257 |
|  | 17225125 |
|  | 12418963 |
|  | 17699753 |
|  | 16000625 |
|  | 17117477 |
|  | 16113653 |
|  | 17644613 |
|  | 16648634 |
|  | 9405416 |
|  | 16283431 |
|  | 11057897 |
|  | 9670954 |
|  | 11525743 |
|  | 8586671 |
|  | 17478078 |
|  | 12650921 |
|  | 11790792 |
|  | 12052834 |
|  | 10446219 |
|  | 14981905 |
|  | 15608632 |
|  | 10521505 |
|  | 16086581 |
|  | 14517246 |
|  | 9211903 |
|  | 12562529 |
|  | 11435459 |
|  | 17591974 |
|  | 16597616 |
|  | 16407310 |
|  | 14991847 |
|  | 10922068 |
|  | 10585392 |
|  | 17583725 |
|  | 10567572 |
|  | 17251057 |
|  | 15591151 |
|  | 12223406 |
|  | 14514687 |
|  | 12912917 |
|  | 12717433 |
|  | 17699782 |
|  | 14729602 |
|  | 14643766 |
|  | 17065197 |
|  | 16771680 |
|  | 12417588 |
|  | 12939259 |
|  | 10946297 |
|  | 15356200 |
|  | 12606754 |
|  | 16557301 |
|  | 11912216 |
|  | 16307741 |
|  | 14657393 |
|  | 10722755 |
|  | 16055088 |
|  | 11971973 |
|  | 10811224 |
|  | 17015681 |
|  | 16135819 |
|  | 16456712 |
|  | 12707267 |
|  | 12808090 |
|  | 11888903 |
|  | 17296730 |
|  | 12391307 |
|  | 10490659 |
|  | 14701702 |
|  | 12857751 |
|  | 17545598 |
|  | 8137421 |
|  | 9851932 |
|  | 12121974 |
|  | 16217033 |
|  | 16676075 |
|  | 14757751 |
|  | 9223324 |
|  | 16434970 |
|  | 7737129 |
|  | 15629131 |
|  | 12167088 |
|  | 15093613 |
|  | 15733859 |
|  | 9733513 |
|  | 12756228 |
|  | 16902157 |
|  | 10756100 |
|  | 15302935 |
|  | 15145937 |
|  | 14704147 |
|  | 9396779 |
|  | 15572149 |
|  | 12148599 |
|  | 11948398 |
|  | 15030387 |
|  | 17187786 |
|  | 16569638 |
|  | 16618812 |
|  | 12646564 |
|  | 16983342 |
|  | 16339571 |
|  | 16699726 |
|  | 9199329 |
|  | 12869386 |
|  | 16005241 |
|  | 17158878 |
|  | 15911351 |
|  | 16581800 |
|  | 16955144 |
|  | 15778501 |
|  | 15059850 |
|  | 12865320 |
|  | 17178870 |
|  | 16777978 |
|  | 12421945 |
|  | 17078934 |
|  | 15456887 |
|  | 12839624 |
|  | 12206715 |
|  | 17287517 |
|  | 12818176 |
|  | 16141073 |
|  | 12359245 |
|  | 16141072 |
|  | 12538493 |
|  | 12689591 |
|  | 15345747 |
|  | 10601313 |
|  | 15569856 |
|  | 16606357 |
|  | 12143039 |
|  | 12734330 |
|  | 12902351 |
|  | 16624865 |
|  | 16760468 |
|  | 16246848 |
|  | 15542843 |
|  | 11865055 |
|  | 9889198 |
|  | 14724588 |
|  | 14638694 |
|  | 16166642 |
|  | 15693750 |
|  | 15192112 |
|  | 17270352 |
|  | 12011047 |
|  | 11781324 |
|  | 15350216 |
|  | 15860507 |
|  | 12810082 |
|  | 16396984 |
|  | 11983683 |
|  | 10799874 |
|  | 16081837 |
|  | 16627484 |
|  | 11525649 |
|  | 16166651 |
|  | 12646240 |
|  | 9661668 |
|  | 12756254 |
|  | 12620934 |
|  | 12225289 |
|  | 11846446 |
|  | 16972261 |
|  | 16043490 |
|  | 10509564 |
|  | 17050683 |
|  | 12878610 |
|  | 17550900 |
|  | 15739188 |
|  | 17255354 |
|  | 12138158 |
|  | 15567863 |
|  | 12023963 |
|  | 12576423 |
|  | 17143273 |
|  | 12897243 |
|  | 7737130 |
|  | 16699462 |
|  | 15225643 |
|  | 14561739 |
|  | 16942465 |
|  | 17301033 |
|  | 10713157 |

---

|  |  |
| --- | --- |
| Organism | Homo sapiens {Organism urn:agi-taxid:9606} |
|  | Mus musculus {Organism urn:agi-taxid:10090} |
|  | Rattus norvegicus {Organism urn:agi-taxid:10116} |
|  | Homo sapiens |
|  | Mus musculus |
|  | Rattus norvegicus |
|  | Danio rerio |
|  | Bos taurus |
|  | Human |

---

|  |  |
| --- | --- |
| Mouse chromosome position | 14 |
|  | 14 B |

---

|  |  |
| --- | --- |
| OMIM ID | 601158 |
|  | OMIM:601158 |
|  | 61411 |

---

|  |  |
| --- | --- |
| Rat chromosome position | 16p16 |

---

|  |  |
| --- | --- |
| Hugo ID | 6881 |
|  | HGNC:6881 |

---

|  |  |
| --- | --- |
| Human chromosome position | 10q11.22 |

---

|  |  |
| --- | --- |
| Swiss-Prot Accession | A1L4K2 |
|  | P45983 |
|  | P45983.2 |
|  | A6P3E4 |
|  | Q7TSJ7 |
|  | G3X8U9 |
|  | Q544A0 |
|  | Q91Y86 |
|  | Q91Y86.1 |
|  | P49185 |
|  | P49185.1 |
|  | B5BTZ5 |
|  | B7ZLV4 |
|  | D3DX88 |
|  | D3DX92 |
|  | Q15709 |
|  | Q15712 |
|  | Q15713 |
|  | Q308M2 |
|  | MK08\_MOUSE |
|  | Q544A0\_MOUSE |
|  | MK08\_HUMAN |
|  | A1L4K2\_HUMAN |
|  | B5BTZ5\_HUMAN |
|  | B7ZLV4\_HUMAN |

---

|  |  |
| --- | --- |
| PIR ID | S71097 |
|  | S71099 |
|  | S43970 |

---

|  |  |
| --- | --- |
| GenBank ID | NC\_000010 |
|  | NR\_136583 |
|  | NR\_136584 |
|  | NR\_136585 |
|  | NM\_001278547 |
|  | NP\_001265476 |
|  | XM\_024448079 |
|  | XP\_024303847 |
|  | NM\_001323302 |
|  | NP\_001310231 |
|  | NM\_139049 |
|  | NP\_620637 |
|  | NM\_139046 |
|  | NP\_620634 |
|  | NM\_001323322 |
|  | NP\_001310251 |
|  | NM\_001323327 |
|  | NP\_001310256 |
|  | NM\_001323331 |
|  | NP\_001310260 |
|  | NM\_001323325 |
|  | NP\_001310254 |
|  | NM\_001323330 |
|  | NP\_001310259 |
|  | NM\_001323326 |
|  | NP\_001310255 |
|  | NM\_001323328 |
|  | NP\_001310257 |
|  | NM\_001323323 |
|  | NP\_001310252 |
|  | NM\_001323324 |
|  | NP\_001310253 |
|  | NM\_001323329 |
|  | NP\_001310258 |
|  | NM\_001323321 |
|  | NP\_001310250 |
|  | NM\_001323320 |
|  | NP\_001310249 |
|  | XM\_024448080 |
|  | XP\_024303848 |
|  | NM\_001278548 |
|  | NP\_001265477 |
|  | NG\_029053 |
|  | AC016397 |
|  | AC074325 |
|  | CH471187 |
|  | EAW93129 |
|  | EAW93130 |
|  | EAW93131 |
|  | EAW93132 |
|  | EAW93133 |
|  | EAW93134 |
|  | EAW93135 |
|  | EAW93136 |
|  | EAW93137 |
|  | EAW93138 |
|  | AA808177 |
|  | AB074280 |
|  | AB451231 |
|  | BAG70045 |
|  | AB451271 |
|  | BAG70085 |
|  | AB451354 |
|  | BAG70168 |
|  | AB451398 |
|  | BAG70212 |
|  | AK094000 |
|  | AK125150 |
|  | AK292523 |
|  | BAF85212 |
|  | AL137667 |
|  | BC130570 |
|  | AAI30571 |
|  | BC130572 |
|  | AAI30573 |
|  | BC144063 |
|  | AAI44064 |
|  | DA159335 |
|  | DQ234352 |
|  | ABB29981 |
|  | L26318 |
|  | AAA36131 |
|  | U34822 |
|  | AAC50607 |
|  | U35004 |
|  | AAC50610 |
|  | U35005 |
|  | AAC50611 |
|  | P45983 |
|  | NC\_000080 |
|  | NM\_001310453 |
|  | NP\_001297382 |
|  | NM\_001310454 |
|  | NP\_001297383 |
|  | NM\_016700 |
|  | NP\_057909 |
|  | NM\_001310452 |
|  | NP\_001297381 |
|  | XM\_006519032 |
|  | XP\_006519095 |
|  | AC154649 |
|  | CH466573 |
|  | EDL24852 |
|  | EDL24853 |
|  | EDL24854 |
|  | EDL24855 |
|  | EDL24856 |
|  | EDL24857 |
|  | EDL24858 |
|  | AB005663 |
|  | BAA85875 |
|  | AB206575 |
|  | BAF48661 |
|  | AB219977 |
|  | BAF69037 |
|  | AI849689 |
|  | AK030767 |
|  | AK032486 |
|  | AK039129 |
|  | AK039254 |
|  | AK043102 |
|  | BAC31460 |
|  | AK044768 |
|  | AK047936 |
|  | AK053062 |
|  | AK085745 |
|  | AK139498 |
|  | AK162915 |
|  | BB619635 |
|  | BC053027 |
|  | AAH53027 |
|  | CD803749 |
|  | Q91Y86 |
|  | NC\_005115 |
|  | NM\_053829 |
|  | NP\_446281 |
|  | AC\_000084 |
|  | AABR07024624 |
|  | AABR07024625 |
|  | AABR07024626 |
|  | AABR07024627 |
|  | AAHX01086374 |
|  | AAHX01086375 |
|  | AAHX01086376 |
|  | CH474046 |
|  | EDL88899 |
|  | EDL88900 |
|  | EDL88901 |
|  | EDL88902 |
|  | AA963835 |
|  | CB782828 |
|  | DV724511 |
|  | EU253566 |
|  | ABX46062 |
|  | FM076271 |
|  | KJ160388 |
|  | AIZ76559 |
|  | L27129 |
|  | AAA42111 |
|  | P49185 |
|  | XM\_011539954 |
|  | XP\_011538256 |
|  | XM\_011539958 |
|  | XP\_011538260 |
|  | XM\_011539956 |
|  | XP\_011538258 |
|  | XM\_011539957 |
|  | XP\_011538259 |
|  | XM\_011539953 |
|  | XP\_011538255 |
|  | XM\_011539955 |
|  | XP\_011538257 |
|  | NM\_002750 |
|  | NP\_002741 |
|  | NC\_018921 |
|  | AMYH02022973 |
|  | AMYH02022974 |
|  | HQ258413 |
|  | ADR83167 |
|  | XM\_006519031 |
|  | XP\_006519094 |
|  | XM\_006519030 |
|  | XP\_006519093 |
|  | XM\_006519033 |
|  | XP\_006519096 |
|  | AC\_000036 |
|  | AAHY01112710 |
|  | AAHY01112711 |
|  | AAHY01112712 |
|  | XM\_006717919 |
|  | XP\_006717982 |
|  | XM\_006717917 |
|  | XP\_006717980 |
|  | XM\_006717918 |
|  | XP\_006717981 |
|  | AC\_000142 |
|  | ABBA01047222 |
|  | XM\_005269973 |
|  | XP\_005270030 |
|  | XM\_005269974 |
|  | XP\_005270031 |
|  | XM\_005269977 |
|  | XP\_005270034 |
|  | XM\_005269976 |
|  | XP\_005270033 |
|  | XM\_005269975 |
|  | XP\_005270032 |
|  | AABR06086523 |
|  | AABR06086524 |
|  | AABR06086525 |
|  | AABR06086526 |
|  | NM\_139047 |
|  | NP\_620635 |
|  | XM\_341399 |
|  | XP\_341400 |
|  | XM\_001056513 |
|  | XP\_001056513 |
|  | NT\_030059 |
|  | NW\_924685 |
|  | NW\_001837974 |
|  | AC\_000053 |
|  | CR614448 |
|  | Q308M2 |
|  | NT\_039606 |
|  | NW\_001030543 |
|  | Q544A0 |
|  | Q7TSJ7 |
|  | NW\_047469 |
|  | NW\_001084711 |
|  | NT\_017696 |
|  | 1082748 |
|  | H3066H10-3 |
|  | 26335518 |
|  | 20986518 |
|  | 20986519 |
|  | XP\_879071 |
|  | 26335519 |
|  | 31418523 |
|  | 20986521 |
|  | 31418524 |
|  | 20986520 |
|  | 20986523 |
|  | 20986522 |
|  | 30316121 |
|  | NT\_039598 |
|  | XP\_869760 |
|  | 26087040 |
|  | 26090526 |
|  | BM940281 |
|  | 493212 |
|  | 493213 |
|  | 127058150 |
|  | 81887723 |
|  | A53063 |
|  | BG081594 |
|  | AAH95795 |
|  | 34877248 |
|  | 22653814 |
|  | 26092533 |
|  | NM\_131721 |
|  | H3073D04-3 |
|  | Q9DGD9 |
|  | NC\_007329 |
|  | 120659944 |
|  | 148692910 |
|  | 148692911 |
|  | 148692906 |
|  | 148692905 |
|  | 148692908 |
|  | 148692907 |
|  | 148692909 |
|  | 2507195 |
|  | 149034131 |
|  | 149034132 |
|  | 9836506 |
|  | XP\_001250450 |
|  | AI316523 |
|  | 149034130 |
|  | 31980670 |
|  | Q502A0 |
|  | 121948371 |
|  | BC095795 |
|  | 7710060 |
|  | XP\_001250405 |
|  | 119613543 |
|  | 119613542 |
|  | 119613544 |
|  | 119613541 |
|  | 119613540 |
|  | NW\_000091 |
|  | 26095546 |
|  | 63100957 |
|  | NW\_001494477 |
|  | 120660426 |
|  | 1346337 |
|  | 119613539 |
|  | 119613538 |
|  | 119613537 |
|  | 119613536 |
|  | 119613535 |
|  | XM\_864667 |
|  | 82192608 |
|  | D730034N23 |
|  | BG068565 |
|  | XM\_001250449 |
|  | 474901 |
|  | BG069159 |
|  | 474900 |
|  | 5730585A15 |
|  | 1463136 |
|  | 1463137 |
|  | BAB11810 |
|  | 1463138 |
|  | 1463139 |
|  | XM\_873978 |
|  | AB030900 |
|  | 1463131 |
|  | 1463130 |
|  | 149034129 |
|  | 6807833 |
|  | 78191533 |
|  | AW909414 |
|  | 4506095 |
|  | BB184171 |
|  | 51468195 |
|  | NW\_001510856 |
|  | 51768363 |
|  | NP\_571796 |
|  | 151175626 |
|  | XM\_001250404 |
|  | BG082181 |
|  | 34877033 |
|  | 34877032 |
|  | 29737804 |
|  | 6141551 |
|  | 6141550 |
|  | NC\_007123 |
|  | 26086964 |
|  | 81894687 |
|  | 20986493 |
|  | NT\_086769 |
|  | NP\_082306 |
|  | BC021788 |
|  | BAE33785 |
|  | AK010301 |
|  | NT\_039474 |
|  | AK144659 |
|  | NC\_000075 |
|  | BAB26834 |
|  | AAH21788 |
|  | NM\_028030 |
|  | BAE25995 |
|  | AK156633 |

---

|  |  |
| --- | --- |
| Swiss-Prot ID | MK08\_HUMAN |
|  | MK08\_MOUSE |
|  | MK08\_RAT |
|  | Q544A0\_MOUSE |
|  | A1L4K2\_HUMAN |
|  | B5BTZ5\_HUMAN |
|  | B7ZLV4\_HUMAN |
|  | D3DX92\_HUMAN |
|  | D3DX88\_HUMAN |

---

|  |  |
| --- | --- |
| Cell Localization | Cytoplasm |
|  | Nucleus |
|  | Extracellular |

---

|  |  |
| --- | --- |
| Ensembl ID | ENSG00000107643 |
|  | ENSP00000378974.4 |
|  | ENST00000395611.7 |
|  | ENSP00000353483.4 |
|  | ENST00000360332.7 |
|  | ENSP00000363297.3 |
|  | ENST00000374182.7 |
|  | ENSP00000363304.1 |
|  | ENST00000374189.5 |
|  | ENSP00000363294.3 |
|  | ENST00000374179.8 |
|  | ENSMUSG00000021936 |
|  | ENSMUSP00000107574.1 |
|  | ENSMUST00000111943.7 |
|  | ENSMUSP00000022504.5 |
|  | ENSMUST00000022504.11 |
|  | ENSMUSP00000107575.3 |
|  | ENSMUST00000111944.9 |
|  | ENSMUSP00000107576.2 |
|  | ENSMUST00000111945.8 |
|  | ENSRNOG00000020155 |
|  | ENSRNOP00000062048.3 |
|  | ENSRNOT00000065216.3 |
|  | ENSP00000378974 |
|  | ENST00000395611 |
|  | ENSP00000353483 |
|  | ENST00000360332 |
|  | ENSP00000363294 |
|  | ENST00000374179 |
|  | ENSP00000363304 |
|  | ENST00000374189 |
|  | ENSMUSP00000107576 |
|  | ENSMUST00000111945 |
|  | ENSMUSP00000022504 |
|  | ENSMUST00000022504 |
|  | ENSMUSP00000107575 |
|  | ENSMUST00000111944 |
|  | ENSMUSP00000107574 |
|  | ENSMUST00000111943 |
|  | ENSRNOP00000062048 |
|  | ENSRNOT00000065216 |
|  | ENSP00000363297 |
|  | ENST00000374182 |
|  | ENSP00000363291 |
|  | ENST00000374176 |

---

|  |  |
| --- | --- |
| MGI ID | MGI:1346861 |
|  | 1346861 |
|  | 1328367 |
|  | 2143015 |
|  | 2145699 |

---

|  |  |
| --- | --- |
| RGD ID | 621506 |
|  | 61411 |

---

|  |  |
| --- | --- |
| Unigene ID | Hs.138211 |
|  | Hs.522924 |
|  | Mm.21495 |
|  | Rn.4090 |
|  | Rn.160441 |
|  | Mm.441782 |
|  | Hs.445864 |
|  | Bt.14050 |
|  | Hs.267445 |
|  | Dr.82505 |

---

|  |  |
| --- | --- |
| Homologene ID | 56760 |

---

|  |  |
| --- | --- |
| Shape | Sickle-vertex |

---

|  |  |
| --- | --- |
| IPI ID | IPI00129682 |
|  | IPI00191803 |
|  | IPI00003145 |
|  | IPI00024672 |
|  | IPI00220305 |
|  | IPI00220306 |
|  | IPI00854859 |
|  | IPI00646929 |
|  | IPI00639865 |
|  | IPI00857698 |
|  | IPI00775848 |
|  | IPI00775931 |
|  | IPI00775897 |
|  | IPI00776030 |
|  | IPI00764183 |

---

|  |  |
| --- | --- |
| Chromosome position | 14 B |
|  | 9 C |
|  | 16p16 |
|  | 10q11.22 |

---

|  |  |
| --- | --- |
| Microarray ID | 104047\_at |
|  | 100391\_at |
|  | A\_52\_P559044 |
|  | A\_65\_P05523 |
|  | 56901\_at |
|  | 84382\_at |
|  | H3066H10-3 |
|  | A\_14\_P122151 |
|  | A\_43\_P15745 |
|  | 2070\_i\_at |
|  | rc\_AI029013\_at |
|  | A\_51\_P422342 |
|  | AK047936 |
|  | A\_65\_P05437 |
|  | 2071\_s\_at |
|  | L27129\_at |
|  | A\_23\_P356152 |
|  | 136576\_r\_at |
|  | 702 |
|  | L26318 |
|  | R637 |
|  | 1420932\_at |
|  | 229664\_at |
|  | OH7871 |
|  | A\_23\_P202540 |
|  | I\_932382 |
|  | 226046\_at |
|  | NM\_016700 |
|  | NM\_002750.2 |
|  | L27129.1 |
|  | A\_14\_P128899 |
|  | 34006\_s\_at |
|  | 210671\_x\_at |
|  | U35005\_s\_at |
|  | 1437045\_at |
|  | A\_24\_P286898 |
|  | 1420931\_at |
|  | A\_51\_P357182 |
|  | A\_52\_P657817 |
|  | 170626\_i\_at |
|  | 226048\_at |
|  | 132248\_r\_at |
|  | 210477\_x\_at |
|  | A\_14\_P107815 |

---

|  |  |
| --- | --- |
| Homo sapiens Chromosome position | 10q11.22 |

---

|  |  |
| --- | --- |
| Ensembl Transcript ID | ENST00000374176 |
|  | ENST00000374182 |
|  | ENST00000374179 |
|  | ENST00000374189 |
|  | ENST00000360332 |

---

|  |  |
| --- | --- |
| ENSEMBL | ENSG00000107643 |

---

|  |  |
| --- | --- |
| FunctionalClass | protein kinase |
|  | MAP kinase |
|  | hydrogen-transporting ATP synthase activity, rotational mechanism |
|  | nucleotide binding |
|  | hydrogen ion transporting ATPase activity, rotational mechanism |
|  | kinase |
|  | protein binding |
|  | protein-tyrosine kinase |
|  | protein kinase CK2 |
|  | JUN kinase |
|  | hydrogen ion transporting ATP synthase activity, rotational mechanism |
|  | protein serine/threonine kinase |
|  | transferase |
|  | hydrogen-transporting ATPase activity, rotational mechanism |
|  | ATP binding |
|  | cAMP-dependent protein kinase |
|  | RNA binding |
|  | nucleic acid binding |

---

|  |  |
| --- | --- |
| Hugo Symbol | MAPK8 |

---

|  |  |
| --- | --- |
| Bos taurus Chromosome position | 28 |

---

|  |  |
| --- | --- |
| Mus musculus Chromosome position | 14 B |
|  | 9 |
|  | 14 |

---

|  |  |
| --- | --- |
| KEGG pathway | Insulin signaling pathway |
|  | Type II diabetes mellitus |
|  | Focal adhesion |
|  | Wnt signaling pathway |
|  | Toll-like receptor signaling pathway |
|  | Colorectal cancer |
|  | Pancreatic cancer |
|  | MAPK signaling pathway |
|  | ErbB signaling pathway |
|  | GnRH signaling pathway |
|  | Fc epsilon RI signaling pathway |
|  | Adipocytokine signaling pathway |

---

|  |  |
| --- | --- |
| State | isoform 4 |
|  | isoform 3 |
|  | isoform 2 |
|  | isoform 1 |
|  | p |

---

|  |  |
| --- | --- |
| Rattus norvegicus Chromosome position | 16p16 |

---

|  |  |
| --- | --- |
| Ensembl Gene ID | ENSG00000107643 |

---

|  |  |
| --- | --- |
| Keyword | map kinase |
|  | RNA |
|  | map kinase kinase |
|  | nuclei |
|  | ERK activator kinase |
|  | PKC |
|  | phosphotransferase |
|  | SAP kinase |
|  | protein phosphatase |
|  | nucleus |
|  | synaptosome |
|  | map kinases |

---

|  |  |
| --- | --- |
| ProteinAtlas NormalTissue | Tissue: adrenal gland; Cell type: glandular cells; Level: Moderate; Expression type: Staining; Reliability: Uncertain |
|  | Tissue: appendix; Cell type: glandular cells; Level: Weak; Expression type: Staining; Reliability: Uncertain |
|  | Tissue: appendix; Cell type: lymphoid tissue; Level: Negative; Expression type: Staining; Reliability: Uncertain |
|  | Tissue: bone marrow; Cell type: hematopoietic cells; Level: Moderate; Expression type: Staining; Reliability: Uncertain |
|  | Tissue: breast; Cell type: glandular cells; Level: Strong; Expression type: Staining; Reliability: Uncertain |
|  | Tissue: bronchus; Cell type: respiratory epithelial cells; Level: Moderate; Expression type: Staining; Reliability: Uncertain |
|  | Tissue: cerebellum; Cell type: cells in granular layer; Level: Negative; Expression type: Staining; Reliability: Uncertain |
|  | Tissue: cerebellum; Cell type: cells in molecular layer; Level: Moderate; Expression type: Staining; Reliability: Uncertain |
|  | Tissue: cerebellum; Cell type: Purkinje cells; Level: Moderate; Expression type: Staining; Reliability: Uncertain |
|  | Tissue: cerebral cortex; Cell type: glial cells; Level: Negative; Expression type: Staining; Reliability: Uncertain |
|  | Tissue: cerebral cortex; Cell type: neuronal cells; Level: Moderate; Expression type: Staining; Reliability: Uncertain |
|  | Tissue: cervix, uterine; Cell type: glandular cells; Level: Strong; Expression type: Staining; Reliability: Uncertain |
|  | Tissue: cervix, uterine; Cell type: squamous epithelial cells; Level: Weak; Expression type: Staining; Reliability: Uncertain |
|  | Tissue: colon; Cell type: glandular cells; Level: Strong; Expression type: Staining; Reliability: Uncertain |
|  | Tissue: duodenum; Cell type: glandular cells; Level: Moderate; Expression type: Staining; Reliability: Uncertain |
|  | Tissue: epididymis; Cell type: glandular cells; Level: Moderate; Expression type: Staining; Reliability: Uncertain |
|  | Tissue: esophagus; Cell type: squamous epithelial cells; Level: Weak; Expression type: Staining; Reliability: Uncertain |
|  | Tissue: fallopian tube; Cell type: glandular cells; Level: Moderate; Expression type: Staining; Reliability: Uncertain |
|  | Tissue: gall bladder; Cell type: glandular cells; Level: Moderate; Expression type: Staining; Reliability: Uncertain |
|  | Tissue: heart muscle; Cell type: myocytes; Level: Moderate; Expression type: Staining; Reliability: Uncertain |
|  | Tissue: hippocampus; Cell type: glial cells; Level: Weak; Expression type: Staining; Reliability: Uncertain |
|  | Tissue: hippocampus; Cell type: neuronal cells; Level: Moderate; Expression type: Staining; Reliability: Uncertain |
|  | Tissue: kidney; Cell type: cells in glomeruli; Level: Weak; Expression type: Staining; Reliability: Uncertain |
|  | Tissue: kidney; Cell type: cells in tubules; Level: Moderate; Expression type: Staining; Reliability: Uncertain |
|  | Tissue: lateral ventricle; Cell type: glial cells; Level: Negative; Expression type: Staining; Reliability: Uncertain |
|  | Tissue: lateral ventricle; Cell type: neuronal cells; Level: Moderate; Expression type: Staining; Reliability: Uncertain |
|  | Tissue: liver; Cell type: bile duct cells; Level: Weak; Expression type: Staining; Reliability: Uncertain |
|  | Tissue: liver; Cell type: hepatocytes; Level: Weak; Expression type: Staining; Reliability: Uncertain |
|  | Tissue: lung; Cell type: macrophages; Level: Moderate; Expression type: Staining; Reliability: Uncertain |
|  | Tissue: lung; Cell type: pneumocytes; Level: Weak; Expression type: Staining; Reliability: Uncertain |
|  | Tissue: lymph node; Cell type: germinal center cells; Level: Moderate; Expression type: Staining; Reliability: Uncertain |
|  | Tissue: lymph node; Cell type: non-germinal center cells; Level: Moderate; Expression type: Staining; Reliability: Uncertain |
|  | Tissue: nasopharynx; Cell type: respiratory epithelial cells; Level: Moderate; Expression type: Staining; Reliability: Uncertain |
|  | Tissue: oral mucosa; Cell type: squamous epithelial cells; Level: Negative; Expression type: Staining; Reliability: Uncertain |
|  | Tissue: ovary; Cell type: ovarian stroma cells; Level: Weak; Expression type: Staining; Reliability: Uncertain |
|  | Tissue: pancreas; Cell type: exocrine glandular cells; Level: Moderate; Expression type: Staining; Reliability: Uncertain |
|  | Tissue: pancreas; Cell type: islets of Langerhans; Level: Weak; Expression type: Staining; Reliability: Uncertain |
|  | Tissue: parathyroid gland; Cell type: glandular cells; Level: Moderate; Expression type: Staining; Reliability: Uncertain |
|  | Tissue: placenta; Cell type: trophoblastic cells; Level: Moderate; Expression type: Staining; Reliability: Uncertain |
|  | Tissue: prostate; Cell type: glandular cells; Level: Weak; Expression type: Staining; Reliability: Uncertain |
|  | Tissue: rectum; Cell type: glandular cells; Level: Strong; Expression type: Staining; Reliability: Uncertain |
|  | Tissue: salivary gland; Cell type: glandular cells; Level: Moderate; Expression type: Staining; Reliability: Uncertain |
|  | Tissue: seminal vesicle; Cell type: glandular cells; Level: Moderate; Expression type: Staining; Reliability: Uncertain |
|  | Tissue: skeletal muscle; Cell type: myocytes; Level: Moderate; Expression type: Staining; Reliability: Uncertain |
|  | Tissue: skin; Cell type: epidermal cells; Level: Moderate; Expression type: Staining; Reliability: Uncertain |
|  | Tissue: small intestine; Cell type: glandular cells; Level: Moderate; Expression type: Staining; Reliability: Uncertain |
|  | Tissue: smooth muscle; Cell type: smooth muscle cells; Level: Weak; Expression type: Staining; Reliability: Uncertain |
|  | Tissue: spleen; Cell type: cells in red pulp; Level: Negative; Expression type: Staining; Reliability: Uncertain |
|  | Tissue: spleen; Cell type: cells in white pulp; Level: Moderate; Expression type: Staining; Reliability: Uncertain |
|  | Tissue: stomach, lower; Cell type: glandular cells; Level: Weak; Expression type: Staining; Reliability: Uncertain |
|  | Tissue: stomach, upper; Cell type: glandular cells; Level: Moderate; Expression type: Staining; Reliability: Uncertain |
|  | Tissue: testis; Cell type: cells in seminiferus ducts; Level: Moderate; Expression type: Staining; Reliability: Uncertain |
|  | Tissue: testis; Cell type: Leydig cells; Level: Moderate; Expression type: Staining; Reliability: Uncertain |
|  | Tissue: thyroid gland; Cell type: glandular cells; Level: Moderate; Expression type: Staining; Reliability: Uncertain |
|  | Tissue: tonsil; Cell type: germinal center cells; Level: Moderate; Expression type: Staining; Reliability: Uncertain |
|  | Tissue: tonsil; Cell type: non-germinal center cells; Level: Moderate; Expression type: Staining; Reliability: Uncertain |
|  | Tissue: tonsil; Cell type: squamous epithelial cells; Level: Moderate; Expression type: Staining; Reliability: Uncertain |
|  | Tissue: urinary bladder; Cell type: urothelial cells; Level: Moderate; Expression type: Staining; Reliability: Uncertain |
|  | Tissue: uterus, post-menopause; Cell type: cells in endometrial stroma; Level: Weak; Expression type: Staining; Reliability: Uncertain |
|  | Tissue: uterus, post-menopause; Cell type: glandular cells; Level: Weak; Expression type: Staining; Reliability: Uncertain |
|  | Tissue: uterus, pre-menopause; Cell type: cells in endometrial stroma; Level: Moderate; Expression type: Staining; Reliability: Uncertain |
|  | Tissue: uterus, pre-menopause; Cell type: glandular cells; Level: Moderate; Expression type: Staining; Reliability: Uncertain |
|  | Tissue: vagina; Cell type: squamous epithelial cells; Level: Strong; Expression type: Staining; Reliability: Uncertain |
|  | Tissue: vulva/anal skin; Cell type: epidermal cells; Level: Moderate; Expression type: Staining; Reliability: Uncertain |

---

|  |  |
| --- | --- |
| LocatorString | pmquery:curcumin+prostate+cancer |
|  | pmquery:curcumin prostate cancer |
|  | gscquery:curcumin+prostate+cancer |

---

|  |  |
| --- | --- |
| State Description | Transcript Variant: This variant (1) is also known as JNK1 alpha1. It encodes the longest isoform (1). |
|  | Transcript Variant: This variant (2) is also known as JNK1 alpha2. It contains extra 5 nt within the coding region, which leads to a translation frameshift, when compared to variant 1. The resulting isoform (2) thus contains a distinct and shorter C-te... |
|  | Transcript Variant: This variant (3) is also known as JNK1 beta 1. It contains a different segment, and extra 5 nt within the coding region, when compared to variant 1. The extra 5 nt leads to a translation frameshift, and results in an isoform (1) tha... |
|  | Transcript Variant: This variant (4) is also known as JNK1 beta2. It contains a different segment within the coding region when compared to variant 1. The translation remains in-frame, and results in an isoform (4) that contains a different internal se... |

---
